# A Sartrean analysis of pandemic shaming

**DOI:** 10.1007/s11097-023-09890-6

**Published:** 2023-01-21

**Authors:** Luna Dolezal, Arthur Rose

**Affiliations:** Wellcome Centre for Cultures and Environments of Health, University of Exeter, Queen’s Building, Queen’s Drive, Exeter, EX4 4QH UK

**Keywords:** COVID-19, Shame, Pandemic shaming, Shame backlash, Shared emotion

## Abstract

In this paper, we analyse the particular phenomena of COVID-19 pandemic shaming. We examine Sartre’s account of the undifferentiated other in the experience of ‘the look’, and his insistence on shame as a foundational relational affect, in order to give a robust theoretical frame to understand how pandemic shaming circulated both online and offline, in targeted and diffuse manners. We focus on two features of pandemic shaming. First, we draw attention to the structural necessity of an audience in acts of pandemic shaming, where the shamer acts on behalf of a community of others, the audience, to perform and enforce a set of standards, values or norms. We turn to the we-experience and collective emotions literature and discuss how the shamer believes themselves to be ‘speaking’ on behalf of a community who share their outrage along with their values. Second, we discuss how the presumption of a collective emotion was frequently mistaken in acts of pandemic shaming, where shaming frequently led to shame backlashes, where the audience revealed themselves not to share the emotion and values of the shamer, consequently shaming the shamer. We argue that Jean-Paul Sartre’s voyeur example is usefully illustrative of the tripartite structure of (1) shamed, (2) shamer and (3) shamer of the shamer that occurs in iterative processes of pandemic shaming, which are accompanied by shaming backlashes. We conclude by reflecting on the socio-historical context for Sartre’s accounts of shame and ‘the look’, namely the German occupation of Paris and Sartre’s experience of the French Resistance movement, and how these yield a particular socio-historical framing that makes evident how the extraordinary pseudo-wartime conditions of COVID-19 rendered atmospheres of distrust and suspicion prevalent.

## Introduction

On the 22nd April 2020, a UK-based Mumsnet user shared a post titled, “To think I shouldn’t be named and shamed for not clapping”. The ‘clapping’ the post referred to was the UK’s regular Thursday night “clap for carers”. Suggested by Dutch UK resident Annemarie Plas, the UK introduced a ritual already prevalent across Europe, where people congregated at their front doors on Thursday evenings at eight o’clock to applaud and cheer National Health Service (NHS) and other frontline workers, as a way to show support and solidarity for those ‘fighting’ on the ‘frontline’. The full post read:

I clapped originally and it was lovely and everyone turned out for it here. Last week, after a rough night with DS [dear son] I fell asleep after he went down and missed the clapping. A post went on our community Facebook group actually naming and shaming me. I was mortified. The post said everyone else turned out and I showed the street up and if I can’t spend a minute showing my appreciation I don’t deserve to use the NHS if I or my family get ill. I ignored it at the time but I can’t get it out of my head it’s really upset me (Aberforthsgoat, 2020).

This case of pandemic shaming seemed to grip the national psyche, with this mum’s revelation making national headlines, while being reported in at least a dozen national and local newspapers. Around the same time, many others came forward to report similar experiences about the Thursday night clap. A *Financial Times* article noted: “Awful stories circulated of people being chastised for missing it, even when the reason was they were NHS shift workers trying to catch up on sleep” (Baggini, 2021).

In the UK, as elsewhere, “pandemic shaming,” that is, publicly naming, blaming and shaming individuals and groups for not properly following the public health rules put in place to curb the spread of COVID-19, or for simply conducting poor pandemic practices (such as hoarding toilet paper or improper hand washing) was a widely reported phenomenon during the early stages of the COVID-19 pandemic, especially during lockdowns. Not only was the media saturated with reports of pandemic shaming, there were numerous articles in US and UK contexts reflecting on the phenomenon, with *The New Yorker* publishing an article in September 2020 declaring that COVID-19 had been the ‘public-shaming pandemic’ (Max, 2020).

In this paper, we turn to Jean-Paul Sartre to analyse the particular phenomenon of COVID-19 pandemic shaming. We examine Sartre’s account of the undifferentiated other in the experience of ‘the look’, and his insistence on shame as a foundational relational affect, in order to give a robust theoretical frame to understand how pandemic shaming circulated both online and offline, in targeted and diffuse manners. In particular we focus on two features of pandemic shaming. First, we draw attention to the structural necessity of an audience in acts of pandemic shaming, where the shamer acts on behalf of a community of others, the audience, to perform and enforce a set of standards, values or norms. To discuss this feature of pandemic shaming, we turn to the we-experience and collective emotions literature and discuss how the shamer believes themselves to be acting on the part of a collective who share their outrage along with their values. Second, we discuss how the presumption of a collective emotion was frequently mistaken in acts of pandemic shaming, where shaming frequently led to shame backlashes, where the audience revealed themselves not to share the emotion and values of the shamer, consequently shaming the shamer. We argue that Jean-Paul Sartre’s voyeur example is usefully illustrative of the tripartite structure of (1) shamed, (2) shamer and (3) shamer of the shamer that occurs in iterative processes of pandemic shaming, which are accompanied by shaming backlashes. Finally, we conclude by reflecting on the socio-historical context for Sartre’s accounts of shame and the look, namely the German occupation of Paris and Sartre’s experience of the French Resistance movement, and how these yield a particular socio-historical framing that makes evident how the extraordinary pseudo-wartime conditions of COVID-19 rendered atmospheres of distrust and suspicion prevalent.

## COVID-19 pandemic shaming

The COVID-19 pandemic was an unprecedented experience that disrupted the very fabric of social, personal and political life. Familiar day-to-day personal and social practices were radically disrupted by public health measures such as social distancing and lockdowns, and we were all thrown into “uncertainty and strangeness” (Aho, 2020, p.2). As such, the norms which governed what behaviour was considered ‘acceptable’, ‘moral’ or ‘ethical’ were in radical flux and this had phenomenological consequences in terms of embodied experiences, social relations and one’s experience of the lifeworld (Dolezal, 2020; Dolezal & Lucas, 2021; Carel, 2020; Aho, 2020; Carel et al., 2020). Public health guidance was put in place to solidify the new rules for social and interpersonal conduct, delineating what was now not only socially acceptable (e.g., keeping two metres away from others at all times), but also legal (insofar as one could incur a fine or penalty for breaking the new rules).

However, in a UK context, public health guidance often lacked clarity and precision, and also changed frequently. For instance, after the first national lockdown commenced, on 23rd March 2020, individuals were ordered to “Stay Home” in order to “Save Lives” and “Protect the NHS”. The clear injunction to “Stay Home”, changed to “Stay Alert” on 10th May 2020 as rules eased. When faced with complaints that the injunction ‘Stay Alert’ was unclear, the British prime minister Boris Johnson encouraged citizens to “apply good, solid, British common sense” to determine how and when to follow public health guidance.^1^ Without clear rules to carve out the normative boundaries of what was ‘acceptable’ or ‘unacceptable’ in particular circumstances or contexts with respect to ‘staying alert’, Johnson instead “invited the public to apply the nebulous and subjective principles of common sense not just in their own decision-making, but to the actions of relatives, friends, neighbours, acquaintances, colleagues and strangers” (Cooper, 2021). As the historian Fred Cooper argues, the reliance on common sense in the UK, in lieu of clear public health guidance, was directly correlated to shame and shaming; it encouraged “informal systems of vigilance, surveillance and recrimination” (Cooper, 2021). In doing so, Johnson also gave individuals license to interpret their personal claims of moral authority as having a collective legitimacy. Pandemic shaming became a means for individuals to police others according to their own ‘common sense’ standards regarding what was ‘right’, ‘acceptable’ or ‘moral’ in the absence of clear and concrete rules, laws or guidelines coming from authorities. It also provided a sense of control and order, while giving some individuals a concrete social role.

Hence, it is not surprising that pandemic shaming was a widespread phenomenon in the UK during the initial stages of the COVID-19 pandemic. With often muddy public health guidance coming from the government, members of the public, often neighbours, were engaged in unofficially policing the behaviour, actions and intentions of others through public, and often social-media facilitated, censure and opprobrium. While ‘curtain twitching’ has long been acknowledged as a part of British social dynamics (Fox, 2005), the unspoken or unofficial surveillance or monitoring of one’s neighbours intensified during COVID-19 lockdowns. So-called pandemic ‘transgressions’ (such as guests staying overnight, illegal indoor gatherings, rule-breaking excursions, etc.) were documented by ordinary citizens presumably looking out for themselves and other concerned members of their community. As the country went into lockdown, physical neighbours began creating correlative virtual groups on Facebook and WhatsApp, with the aim of staying in touch and helping each other out during this period of extreme isolation. These social media and communication platforms became frequent sites of neighbourly shaming, where individuals ‘called out’ neighbours for their poor pandemic practices, as illustrated by the Mumsnet post quoted above. In addition to these informal shaming acts, during the UK’s first lockdown, police were inundated with thousands of complaints and reports about people allegedly breaking lockdown rules, where neighbours were routinely informing on each other to the authorities.

Pandemic shaming was facilitated by the formation of “techno-social niches”, to use Krueger and Osler’s term, that involve both online and offline space and are “permeated by the physical and/or virtual presence of others” (Krueger & Osler, 2019, p.212). Beyond the concerns of particular neighbourhoods or communities, pandemic shaming proliferated more widely through online platforms, where hashtags such as #selfishpricks and #covidiots became shorthand ways to call out poor pandemic practices across a wide range of social contexts. Indeed, the #covidiot phenomena perhaps crystallises the essence of pandemic shaming. From March 2020, invoking the neologism ‘covidiot’ (a contraction of covid + idiot) especially on social media, became an instantaneous means to attempt to police the behaviour of individuals through naming, blaming and shaming (Cooper et al., 2023). In this way, pandemic shaming was enacted, or made possible, through our contemporary habitation of what Krueger and Osler call “blended spaces”, where our lifeworlds, activities, communities, communications and actions span, or are a blend of, both offline and online spheres (Krueger & Osler, 2019). As lockdowns plunged us increasingly into online spaces, these spaces, especially social media platforms, became the sites for the airing of personal and local grievances that occurred offline, in other words, for the circulation of shaming and censure. However, as the Mumsnet example illustrates, pandemic shaming did not just land on the so-called ‘idiots’ who ignored or flaunted public health rules at the expense of potentially actually harming others, but also became a means to enact a moralising surveillance on those deemed to not be behaving with pandemic propriety within a particular community or neighbourhood, or those who failed to enact ritualised pandemic behaviours (such as participating in Clap for Carers).

## The phenomenology of shame and shaming

While acts of pandemic shaming may have sometimes led to the experience of shame, it is important to note that shame and shaming are structurally distinct. Shame is commonly characterised as a self-conscious emotion of self-assessment that causes the subject to feel discomfort and anxiety at the thought of how they are seen and judged by others (Zahavi, 2014; Sartre, 2018). Shame is an experience that involves an awareness of the self, of one’s mishap or transgression, and also crucially, an awareness of how another, or others, see (and judge) the self.^2^ As Sartre writes: “Shame … is the *recognition* that I really *am* this object that is looked at and judged by the Other” (Sartre, 2018, p.358). As a result, shame is characterised as a fundamentally “self-conscious evaluative emotion” (Draghi-Lorenz et al., 2001, p.270). Fundamental to self-conscious evaluative emotions is the ability to compare oneself, or one’s behaviour, actions or circumstances, with internalised social norms, and then respond to the outcome of this comparison or evaluation. These sorts of emotions also involve the capacity to regard the self as though from the perspective of an ‘other’, or an external observer (Draghi-Lorenz et al., 2001, p.270). As Sartre puts it: “Thus shame is the unitary apprehension of three dimensions: ‘*I am* ashamed of *myself* before *the Other*’” (Sartre, 2018, p.393). In this way, shame is perhaps more accurately to be understood as a “self-other-conscious” emotion (Reddy, 2008, p.224). It involves reflexive awareness of oneself and a necessary triangulation of experience that involves the self, the other and social norms.

The experience of being seen by others and being a shamed object for others is central to Jean-Paul Sartre’s phenomenological ontology through his well-known account of the ‘the look’. For Sartre human existence is characterised by “the constant possibility of my *being seen* by the Other” (Sartre, 2018, p.352). He famously argues that the origin of reflective self-consciousness is located in the perceptual encounter with the ‘other’ (Dolezal, 2012). The relation of the other’s ‘look’ is constitutive: “It suffices that the Other should look at me, to make me what I am” (Sartre, 2018, p.359). Crucially, the look of the other, for Sartre, is never neutral. Instead, it is always value-laden. My ability to ‘see’ myself is afforded by the other’s look which gives me an “outside”, as Sartre puts it (Sartre, 2018, p.392), and this occurs through a moment of shame: I suddenly see myself as the other sees me (as an object of their judgemental regard) and as a result I feel shame. As Sartre puts it: “Shame is shame of *oneself*; it is the *recognition* that I really *am* this object that is looked at and judged by the Other” (Sartre, 2018, p.358). Hence, shame in Sartre’s account has ontological significance, it reveals both “all the structures of my being” and also the “indubitable presence” of the other (Anderson, 2021).

Sartre’s account of ‘the look’ and its inherent shaming potential is illustrated by the oft-cited scene of the voyeur in *Being and Nothingness* (Sartre, 2018, p.355–357). Kneeling at the keyhole, overcome by jealousy, the voyeur is spying on their lover. In the moment of spying, the voyeur is completely caught up in the act, and does not have a sense of the external meaning of this act of spying. An emotional anxiety, namely their jealousy, drives their behaviour and organises their actions and interactions. Sartre, adopting the first-person perspective of the voyeur, writes, “I *am* this jealousy; I do not know it. I could learn of it from the worldly equipmental structure only if I were contemplating it rather than creating it” (Sartre, 2018, p.356). To the spectacle through the keyhole that is proposed as ‘to be seen’, their actions turn; before the other arrives, there is no transcendent view that might “confer on the acts the character of something given, to which a judgement might be applied” (Sartre, 2018, p.355). Hearing footsteps behind them, the voyeur is suddenly made aware of how what they are doing appears from outside, they are *seen* by the other and through that seeing, shame arises. They suddenly realise and know that they are a voyeur; that they are spying, and so on. Instead of being lived through unselfconsciously, their actions and appearance become laden with value, conditioned by the judgemental attitude inherent in the other’s look. In short, hearing the footsteps behind them in the hallway, the voyeur is caught-in-the-act and feels the flush of shame.

Sartre’s voyeur example also illustrates the diffuse and undifferentiated nature of the look of the other. Sartre is at pains to illustrate that shame can arise when one is alone, that the ‘other’ is simultaneously *everywhere*, but *nowhere* in particular. After hearing the footsteps behind them, the voyeur straightens up and realises that there is in fact no one there and that they were mistaken about the physical presence of another person (Sartre, 2018, p.377). Indeed, Sartre makes it very clear that while the look “is manifested *most often* by the convergence of two eyeballs towards me” (Sartre, 2018, p.353) the empirical presence of another person is by no means necessary for the look to have its constitutive effect. As Sartre writes, the look “can show itself just as well in a rustling of branches, a sound of steps followed by silence, a half-open shutter, a slight movement of a curtain” (Sartre, 2018, p.353). In this way, Sartre argues, “The Other is present to me everywhere, as that through which I become an object” (Sartre, 2018, p.381). Sartre’s account of ‘the look’ offers useful resources to understand how being seen and judged by others can occur not only in face-to-face encounters, but also in indirect and mediated experiences such as curtain twitching and via online spaces where there may be an undifferentiated, unspecific or merely suggested sense of being ‘seen’ by another or others.

If we attempt to map an incident of pandemic shaming onto Sartre’s account of shame through the voyeur example, then in a straightforward reading, the footsteps in the hallway take the position of a shamer (one who witnesses the so-called transgression and instigates the act of shaming), while the voyeur represents the one who is or feels shamed. When we attempt to apply this account to our Mumsnet example, however, an immediate problem arises. It would position the mum who fell asleep and missed an evening of Clap for Carers as the voyeur and her neighbour as the footsteps in the hallway. But, since the footsteps are taken to be an imagined presence, the only being-for-itself involved is the person feeling shame: the voyeur. No adequate correlative to the shamer, as a being-for-itself, exists. The problem, of course, is that Sartre’s account is not one of active *shaming*, but one that illustrates the structural possibility of shame in social relations, where a concrete other is not necessary for shame to arise. In short, Sartre’s voyeur example is not an account of *shaming*, but an account of *shame*.

At this stage, it is important to differentiate *shame* and *shaming*. In contrast to shame, shaming is not a first-person shame experience, as described above, where one feels seen and judged by an ‘other’ or ‘others’ (whether they are present, potential, internalised or imagined). However, the aim of shaming is usually to induce a first-person experience of shame in the one who is the recipient of the shaming act, but this is by no means a necessary outcome. Martha Nussbaum takes shaming to be a stigmatising judgement, where an individual or a group judges and condemns another or others for transgressing or failing to live up to an ideal or norm that is shared by a community, by society or by a cultural or political grouping (Nussbaum, 2004, pp.184–86). In this way, shaming is an attempt by the shamer to coerce or punish the shamed through using a powerful affective force, namely shame, as a means to motivate conformity to particular norms or standards. As Creed et al. note, “shaming attempts are situated, purposive uses of episodic power to induce compliance with institutionalized community prescriptions” (Creed et al., 2014, p.284). Feeling shame is a powerful motivator for conformity, as shame carries “implicit or explicit threats of temporary ostracism, or even the permanent sundering of social bonds” (Creed et al., 2014, p.284). In other words, individuals who are shamed, and who experience shame as a result, feel acutely that their social standing and hence their social bonds are under threat and that their sense of belonging may be compromised. The anguish and emotional pain of shame is about the fear or anxiety that one may be rejected, shunned or ostracised, that one is vulnerable to losing a sense of belonging (Dolezal, 2017). Shaming is an attempt to instil this anxiety in an ‘other’ or ‘others’ by threatening their social bonds through a public performance where they are deemed or labelled ‘shame-worthy’ or ‘shameful’. It should be noted that attempts at shaming may result in shame. However, they may also sometimes fail to induce shame. The power to shame and to be shamed is intimately tied up with the distribution of social power and one’s relative social position, where those with higher levels of social power are more likely to be able to “punch down” and shame those with less power or privilege,^3^ even if social media affords opportunities for individuals with less social power to “punch up” (O’Neil, 2022). For shaming to provoke the first-person experience of shame, it seems to be necessary for the individual being shamed to have some stake in the norms that are being transgressed or violated, but this is not always the case.^4^

Importantly, not all shaming prioritises the instilling of the first-person experience of shame in an ‘other’. Shaming can also be about the performance of one’s values and standards in the service of reinforcing in-group belonging through creating ‘others’ or scapegoats. Or shaming may be deployed to motivate changes in behaviour through threatening or compromising reputations, simply by marking an other or others out as recipients of shaming, or worthy of shame (Jacquet, 2015, p.9). As Creed et al. note:

Shamers are interested members of a community who act as institutional guardians and have cognitive, emotional, and/or moral commitments to existing institutional arrangements, including definitions of acceptable behavior and established patterns of social relations. Shamers actively police the boundaries of acceptable behavior using episodic shaming to alert transgressors that social bonds linking the transgressors to the community are at risk (Creed et al, 2014, p.280).

In judicial and official punitive contexts (such as law enforcement), shaming is often presumed to serve a pro-social function; the idea is that through a moment of being shamed one recognizes that one has fallen short of the standards of one’s community and makes positive changes to bring oneself back in line with the community’s values, norms and mores, perhaps after a period of diminished social standing. This “reintegrative shaming” serves a pro-social function, reinforcing rather than severing belonging (Braithwaite, 1989).

Of course, the suggestion that shaming has this pro-social function is possible because shame itself has this reintegrative and pro-social potential. In keeping with the work of Erwin Straus and Max Scheler, Zahavi reminds us that shame includes not just the painful emotion in a moment of exposure to the other (as per Sartre), but also the perception of what is improper or disgraceful (Zahavi, 2014, p.214), a perception once described in English as “shamefast” and for which only the rather anodyne cousin, “modesty,” remains. This leads Zahavi to observe that “rather than being inherently debilitating” as Sartre’s account suggests we are always negatively objectified by the other, “shame might also play a constructive role in moral development, and not only because it can aid socialization by promoting social conformity, but because it can disrupt my self-complacency, modify my self-understanding, and in the long run motivate me to reorient my way of living” (Zahavi, 2014, p.215). While shame and shaming have pro-social potential, it should be noted that shaming is extremely unpredictable and often backfires. While shaming may occassionly lead to positive change, it is more likely to cause harm, perhaps exacerbating existing inequalities and injustices, or provoking defensive reactions which may harden negative patterns of behaviour (Walker, 2014, p.52).

Not every moment of shame is simply about being judged and objectified by others in an intersubjective moment as Sartre’s account seems to suggest. Shame, as noted above, is connected to the broader socio-political milieu, where it carries a sense of what is improper with respect to prevailing social norms. The negative correlative of this insight is, of course, that Sartre makes no room in his account for the ways that shaming, as a moral practice, might be brought to bear on me as an action of the other. In short, Sartre’s account of shame in *Being and Nothingness* does not consider shaming and how the normative values of a community can be instilled through shared intentions and collective emotions. In fact, as Zahavi points out, Sartre’s account of intersubjective relations is “ultimately conflictual” and denies the possibility of a “we-subject” that may have shared intentions (Zahavi, 2001, p.139).

Acts of shaming by an individual presuppose a shared intention, or “we-intention” (Zahavi, 2015), where the shamer is acting with, or on behalf of, a community of others who share their values, whether this is explicit (there are multiple shamers in explicit agreement) or implicit (I act alone, but believe and feel that my community of others is agreement and feels the same outrage). We-intentions are not just necessary for acts of shaming, but are fundamental to the possibility of having shared norms and values in general. As Zahavi notes, “the capacity to have we-intentions is fundamental to human social life and social (e.g., institutional) reality. It is a crucial prerequisite for the creation and maintenance of social norms” (Zahavi, 2015, p.84). Above this fundamental level of shared experience where there is a common social landscape of norms and values, in an act of shaming, the shamer believes there is some level of “we experience” through “emotional sharing”, where others in their community share their outrage against the one who is being shamed (Zahavi, 2015, p.90). Presumably the neighbour on the doorstep assumed her community was in agreement with her condemnation of the mum who missed the clapping. Her online post had a twofold function: it attempted to incite shame in the mum; and it publicly performed the (presumed) shared values of the shamer’s community in order to reinforce them.

## Shame backlashes

However, the assumption of emotional sharing and of a we-experience in acts of pandemic shaming was often faulty. What has been interesting about pandemic shaming is how often such interventions have backfired. Acts of pandemic shaming were often, if not almost always, accompanied by shame backlashes. In our Mumsnet example, far from endorsing the original Facebook post, the newspaper stories, online articles and social media posts that proliferated about the incident all came down against the self-appointed moral police-person; in short, they shamed the shamer. We don’t have an account of what happened to them, but it seems reasonable to surmise that their subsequent experience was not satisfaction in having played a constructive role in moral development, but rather a parallel moment of “being-seen-by-the-Other”: the footsteps in the hall come to be a judgemental gaze that itself confers shame on the shamer.

Shame backlashes have become a commonplace occurrence in the online world and point to the general volatility and unpredictability of online shaming and the more general “call out culture” that has been facilitated by the supposedly democratic spaces afforded by social media. These shame backlashes, as Karen Adkins notes, are “more than simply the refusal of a judgment of shame (I am not embarrassed or stigmatized by what I did), but an aggressive redirection of shame (you, the shamer, should feel shame for trying to diminish me. Your attempt at shaming reveals your lack of character)” (Adkins, 2019, pp.76–77). If we see the central moment of shame in Sartre’s voyeur example as simply the voyeur’s response to the footsteps in the hall, then we miss an important structural element of shame that circulates in the blended spaces of our contemporary social world. Namely, the audience. As Adkins argues:

Nussbaum, as with many other theorists of shame, takes for granted the comprehensibility and acceptability of shame: that there is a shared body of values among shamer, shamed, and audience, and thus the shaming will be effective. This treatment of audience both minimizes its importance to a theory of shame, and also reduces its role to a passive one; the audience exists merely to amplify the judgment being passed (Adkins, 2019, p.78).

Most pandemic shaming was not of the judicial or formally punitive kind that concerns Nussbaum in her discussion of shaming punishments, where it is presumed that there is a shared and agreed upon body of values by which the shaming is enacted. Instead, most pandemic shaming was informal and social. They were acts by individuals from the community taking upon themselves to assert their moral codes, to police the moral boundaries of good/bad or appropriate/inappropriate pandemic behaviour. This informal and social shaming is, as Adkins notes, “disputable” and “presents risks” as the shamer is claiming to be “epistemically and socially empowered” to speak on behalf of a community, but may not necessarily be seen by others (i.e., the audience) to be so (Adkins, 2019, p.82).

This demonstrates a problem raised by Stanley Cavell that, “when speak[ing] for the others with whom you consent to association”, “we do not know in advance what the content of our mutual acceptance is, how far we may be in agreement” (Cavell, 1999, pp.27;28). Cavell suggests that even the identity of “these others […] for whom you speak and by whom you are spoken for, is not known a priori, though it is in practice generally treated as given” (Cavell, 1999, p.27). And so, because there is no agreement on what “we” say, or even who this “we” is, to speak (or shame people) on behalf of others, risks “the rebuff — on some occasion, perhaps once for all — of those for whom you claimed to be speaking; and it means risking having to rebuff —on some occasion, perhaps once for all — those who claimed to be speaking for you” (Cavell, 1999, p.27).

This richer account of the fraught nature of shame, shaming and ‘speaking for others’ can help us to refigure Sartre’s voyeur example as a spectacle. Here, the relation between the voyeur and their lover via the keyhole, introduced by Sartre for its explicative value, becomes a demonstration of active shaming that plays out to an audience (the footsteps). As this audience can influence the actors by booing or cajoling them, it is no longer passively in agreement but becomes instead a key agent in the shaming dynamic. As Adkins notes, “Audiences of shaming matter because shaming is a hybrid act. It is an epistemic as well as an ethical act (I judge this behavior as unworthy of this stated community norm), and to be effective relies upon a friendly social reception on both accounts” (Adkins, 2019, p.79). While moralising shaming is not explicit in Sartre’s account of shame, the central example of the voyeur offers a surprisingly apt structure for the iterative, blended spaces of pandemic shaming. Indeed, a much closer analogue to the position of the voyeur is the curtain-twitching neighbour who is spying on the beleaguered mother and reporting her. What is not frequently acknowledged or reflected upon in the Sartre literature is the fact that the voyeur is also an ‘other’ who is ‘looking’; they, after all, are caught in the act of spying; they are looking through the keyhole, driven by their jealousy they are there to ‘call out’ their lover.

If, in our opening example, the voyeur maps on to our curtain-twitching neighbour and the lover behind the keyhole maps on to our beleaguered napping mum who is being spied on, then the footsteps in the hall (the undifferentiated ‘other’) becomes the audience of the shaming. In our example, there is not just the mum and her nosy and judgemental neighbour, but also an additional layer of looking, judging and shaming, that is, the much broader censure of this shaming incident by the local and national newspapers who carried the story, and the general public who in turn used social media to shame the shamer. In short, this instance of pandemic shaming, as like many others, also involved the shaming of the shamer, or a “*shame backlash* against the shamers” (Adkins, 2019, p.76).

This three layered structure, (1) the object of shame, (2) the shamer and (3) the shamer of the shamer, aligns neatly with Sartre’s three figures: the lover, the voyeur and the footsteps. It also captures the dynamics of pandemic shaming that arose during COVID-19 lockdowns, where intangible and remote audiences often had the time and interest to follow the vicissitudes of a shame-shamer interaction. While all acts of shaming are never neutral, but always “reliant … on a friendly audience” (Adkins, 2019, p.77) who will not challenge the authority, credibility or legitimacy of the shamer, the way in which pandemic shaming circulated both online and offline was facilitated by the way in which contemporary shaming has been transformed in the blended spaces of social life. Namely, the online audience is frequently undifferentiated, diffuse and anonymous (where ghostly footsteps become an apt image for this intangible but ever-present audience).

As such, when it comes to acts of shaming, the affective scaffolding that attends the blending of online and offline spaces may very well preclude attempts by autonomous individuals to assert moral standards on a community’s behalf, unless they have enough loyal followers to drown out dissident voices, or the financial resources to harness trolls or bots to do the same thing. As Osler and Szanto observe of interpersonal atmospheres at a party, “the festivity of the party is not located in one individual, it is not just one person’s happiness, rather the festivity arises from and between those present” (Osler & Szanto, 2021, p.166). Importantly, it is beyond the means of any one person to control the atmosphere of a social media storm; at best, they may influence it, depending on the size of the space and the number of followers they have. Equally, as Osler and Szanto note, “we might simply get the tone of the atmosphere wrong. In the same way that one might mistake another’s grimace for a smile, our experience of interpersonal atmosphere can be wrong” (Osler & Szanto, 2021, p.167). When one announces this mistake publicly, one may receive some correction. The difference, here, is that the voyeur, in getting the mood, values or emotions of the community wrong, is not simply embarrassed by their friends correcting them, as in an interpersonal atmosphere; rather they are themselves publicly shamed.

This may explain why, when pandemic shaming was widely reported in media outlets in the UK and US, most of this reporting shamed the shamer, questioning the authority, credibility and legitimacy of pandemic shamers. When the curtain-twitcher finds themself on social media, performing their role as social police-person, they are using these platforms like Sartre’s voyeur, who, driven by emotional anxiety (in their case, jealousy), uses the door and the keyhole, as equipment to serve their moral regard. Like footsteps in the corridor, the unintended audience response to this performance of moral regard produces essential modifications in the curtain twitcher’s structures. Indeed, here it seems entirely in keeping with other famous instances of online, public shaming that begin from an ill-considered social media update: if the effects on the individual are pernicious and long-lasting, in part the trauma comes from the message’s intended meaning being obscured, almost entirely, by the way it is taken up by its audience.

Given the amplified effect of these blended spaces, we might then reassess another standard line in criticism of Sartre, which complains about the reductive negativity in his look. Martin Jay criticises Sartre’s account of the look for being “described in the most frighteningly negative terms” (Jay, 1993, p.276), where intersubjectivity and self-other relations are always marked by suspicious and objectifying encounters. Sartre’s world is comprised of a “universe of threatening gazes” always resulting in “shame” as Jay puts it (Jay, 1993, p.289). Of course, not every encounter in self-other relations is marked by shame or by objectifying judgemental gazes. Zahavi writes: “The nature of the look can … vary enormously” (Zahavi, 2014, p.223). For example, Marjorie Grene suggests that there are many situations when the look is not threatening; consider the “rare but still indubitable experience of mutual understanding, of the reciprocal look of peers; or the look of mother and infant, where the one protects and the other is protected” (Grene, 1983, p.154). Even if we concede to Grene that Sartre’s account does not adequately differentiate between looks, and to Jay that Sartre could lighten up, the generally toxic environment of online shaming probably demands an account built less on empathy than on mutual suspicion, ready-made contempt and casual trolling. Indeed, it seems that Sartre’s Hegel-inflected account of shame as an ontological self-other conflict is entirely consistent with the way that we can be taken up by the affective scaffolding of others in our internet-based communications.

A social media landscape can sometimes be experienced as a ‘universe of threatening gazes’. If being-on-the-internet is always a being-before-others, or a being before an ‘audience’, to use Adkins term, then we are always at risk, in those techno-social niches that we share with others, of having our expressions taken up and re-interpreted or re-worked by those others. In other words, the shamer is always at risk of being shamed. When this reworking leads to social exclusion, then we can see how a Sartrean account of shame becomes particularly pertinent.

## The wartime logics of COVID-19 and the French occupation

However our aim is not simply to ‘apply’ Sartre to pandemic shaming, but rather to show a deeper relevance of his account as a result of the particular socio-historical circumstances from which it arose. We now historicize Sartre’s account of shame, suggesting that his examples of rustling bushes and twitching curtains, far from lacking any significance in themselves, betray a lurking paranoia entirely consistent with Paris under Occupation, as it was in 1943 when *Being and Nothingness* was published. Although such references can, of course, be threaded together, we turn to a more compelling description of the atmosphere under which the book was written: Sartre’s 1945 essay, “Paris under Occupation” (Sartre, 1998). In emphasising the bathetic, day-to-day anxieties of the German Occupation, the essay bridges the exceptional state of wartime occupation with the comparatively more banal condition of COVID-19 lockdowns, under which pandemic shaming thrived.

The conditions of COVID-19 lockdowns in the UK were often likened to living through wartime conditions (Rose & Dolezal, 2020), where the WWII “blitz spirit” was frequently invoked as a means to get through the personal and social difficulties that arose (Jones, 2020). Yet, as work on health-related war metaphors has shown, this language is “ironic, unfortunate and unnecessary” (Nie et al., 2016, 9). Writing in response to HIV research, Nie et al. observed not only that the aims of healthcare (healing) and warfare (killing) directly contradict each other, wartime metaphors are unfortunate because they “can inadvertently further stigmatize patients, inflict additional suffering on them, and endorse the legitimacy of war and violence in social and political life” (Nie et al., 2016, 9). Such metaphors, Franziska Kohlt has observed, played a crucial role in shaping the narrative understanding of COVID-19 as analogous to a wartime situation. Importantly, the short-term benefits of war rhetoric in raising public awareness and the willingness to conform to health protocols “can in the long term lead to increased anxiety, and damage trust in those communicating science in public” (Kohlt, 2020, 18). Accordingly, one should be wary of introducing uninterrogated wartime analogies in ways that reinforce the assumption that epidemics are best understood as wartime conditions.

Nevertheless, the comparison between lockdown and occupation does prove salient and not simply as the reality that Sartre lived through while writing *Being and Nothingness*. Historicizing its accounts of shame and the look as products of the Occupation’s atmosphere reminds us of the conditions in which Sartre’s heightened sense of being observed emerges (Kitchen, 2013). We have already recalled the moment when Sartre declares: “a look is manifested *most often* by the convergence of two eyeballs towards me. But it can show itself just as well in a rustling of branches, a sound of steps followed by silence, a half-open shutter, a slight movement of a curtain” (Sartre, 2018, p.353). To further illustrate how ‘the look’ can be dislodged from an empirically present set of eyes, he continues: “In the course of a *coup de main*, what the men crawling in the bushes apprehend as a *look to be avoided* is not two eyes, but an entire white farm standing out against the sky at the top of a hill” (Sartre, 2018, pp.353–354). In *Being and Nothingness*, the undifferentiated ‘other’ is over and over again portrayed as a menacing military presence: “it is not certain that the enemy soldiers are at this moment watching through its windows” (Sartre, 2018, p.375). It has been acknowledged that Sartre’s turn to military examples, where a paranoid, sinister and threatening atmosphere prevails in his discussions of the look and relations with others, is due to the pervasive influence of the German Occupation of France during World War II, during which time Sartre was writing *Being and Nothingness*.^5^

In “Paris Under Occupation”, Sartre attempts “to try and show in what way Parisians *experienced* the occupation” (Sartre, 1998, p.2). In this essay, Sartre is at pains not to sensationalise the occupation, but instead to describe how it was a “daily affair” (Sartre, 1998, p.2), where day-to-day life continued, but under conditions of duress, with restrictions and within an atmosphere of horror, shame, suspicion and distrust; conditions strikingly similar to COVID-19 lockdowns. Of course, these were not parallel events; the truth of the Occupation was that people were specifically targeted by the German army and their informants, circumstances that cannot be compared to the movements of a virus. What is germane to our analysis is the way that Sartre describes this experience for those who were not targeted, but for whom the occupation was endured as a day-to-day reality.

First, he dispels the illusion that the German soldiers themselves appeared as a threatening presence. He recalls “the same daily necessities” and “the same collective currents” that frequently brought them together, from squeezing into a subway to bumping into each other at night (Sartre, 1998, p.3). Their identity as “an enemy” became blurred as soon as German soldiers and French citizens were no longer separated “by a line of fire” (Sartre, 1998, p.4). This did not mean that there was no enemy: Sartre is clear that there was, “but he didn’t have a face” (Sartre, 1998, p.4). This enemy seized men in the dark and made them disappear, swallowing them in silence. Paris itself seemed to have “hidden holes” through which people disappeared “as if seized by an internal and undetectable hemorrhage” (Sartre, 1998, p.4). Sartre attributes the disappearances to “a kind of living and impalpable tar that blackened everything, including the light” (Sartre, 1998, p.4). The steps heard outside are *theirs*; the outside a no-man’s land “inhabited by them only”; the doors of houses threaten to open to let *them* in (Sartre, 1998, pp.4–5). Even when unthought, this presence is felt in household objects, which “were less our own, stranger, colder, more public somehow” (Sartre, 1998, p.5). Crucially, Sartre explicitly links this alienation to the look, which he imagines “violating the intimacy of our homes” (Sartre, 1998, p.5). The Occupation, for Sartre, became “a perpetual co-existence with a phantom-like hatred and an enemy who is too familiar to be hated successfully” (Sartre, 1998, p.5).

There is plenty to unpack here; the looks, the uncanniness of the objects, the sense of a retreating agency, all these remind us of the pinioned voyeur whose shame experience demands only the implied presence of an ‘other’ to find themselves objectified in an imagined gaze. But it also specifies a context in which norms are in radical flux. As in the pandemic, Sartre’s Occupation involved a disruption of social norms and habits. A new set of norms and habits were imposed even as prior practices were rendered suspect. Further, these new practices were explicitly contingent and liable to be changed at any point, as circumstances deemed necessary (e.g., the appearance of new virus variants, or the changing whims of the occupying presence). Sartre locates this interruption of habit in the physical absences of the city: “Paris was dead. No more cars, no more pedestrians … the skeleton of the city remained, fatuous and immobile and too big for us: the streets stretched out endlessly and were too wide, the distances too great, the perspectives too vast” (Sartre, 1998, p.5–6). Anyone recalling photographs of major metropolitan centres during lockdowns would find this passage resonant. However, perhaps more crucial to our point are the two existential responses that Sartre identifies as being triggered by these expansive absences in social spaces. The first provokes a loss of a sense of future possibility, stripping individuals of their projects and purpose: “Parisians stopped projecting their future beyind themselves and, at the same time, can no longer recognize the future of others … the occupation stripped people of their future” (Sartre, 1998, p.8). The second attributes this loss of a future to an increased dependency on the decisions of others (i.e., the occupiers or the English) for whom “we were only an object” (Sartre, 1998, p.7). The physical absences, then, play a role in generating a collective sense of lost futures, which, in turn, provokes the sense of being an object for others that Sartre identifies as constitutive in the shame experience. Sartre concludes, “Hence we felt out of the game. We were no longer participating in the war and because we no longer understood it, we felt ashamed” (Sartre, 1998, p.8).

The Occupation in Sartre’s description presents surprisingly parallels to experiences of the UK’s COVID-19 lockdowns, where against a backdrop of political and media discourse that was saturated with military metaphors (e.g., “health workers [were] “servicemen” on the “frontline” “battling” “an invisible enemy” (Rose & Dolezal, 2020)), individuals had their daily lives curtailed, much like the Parisians Sartre describes who “stayed home or confined themselves to their neighbourhood” (Sartre, 1998, p.6). Certainly, it seems, again, that attempting to parallel Occupation experiences with those of a pandemic lockdown risk aggrandizing the latter and trivializing the former. Except that Sartre himself acknowledges that “one shouldn’t imagine it as a vivid and violent emotion. […] *We continued to live* […] we could work, eat, talk, sleep sometimes even laugh […] the horror seemed to be outside, within things” (Sartre, 1998, p.5).

Hence, rather than likening the condition of COVID-19 lockdowns in the UK to wartime, it would be far more accurate to compare lockdown conditions with that of a military occupation, such as Sartre describes (e.g., restrictions in daily life, a confinement to one’s home, a diffuse and often invisible enemy, the continued mundanity of day-to-day life). Under the affective climates of occupation, it is not surprising that Sartre’s world of social relations in *Being and Nothingness* is a ‘universe of threatening gazes’ nor is it surprising, if we read *Being and Nothingness* through a historicized lens, that fear, vulnerability, shame and defenselessness are the consequences of the look: enemies and informants are lurking behind curtains and bushes. Sartre writes: “What I grasp immediately when I hear the branches breaking behind me is not that *someone is there*, but that I am vulnerable, that I have a body that can be hurt, that I am occupying a place and that I cannot in any circumstance escape from the space in which I am, defenceless - in short that I am *seen*” (Sartre, 2018, p.355). Shame is implicated with this defenselessness: the occupied subject in Paris “lived in despair and shame” (Sartre, 1998, p.2) as Sartre describes it, as a result of their subjugated and vulnerable state: afraid of the enemy, afraid of informants. Indeed, the parallels in circumstances and atmosphere between the German occupation of Paris and the UK’s COVID-19 lockdowns suggest that Sartre’s accounts of shame, the look and the voyeur may usefully describe the multi-layered phenomena of pandemic shaming because the affective climates of these particular social worlds led to parallel emotional landscapes. The increased vulnerability that accompanied subjects during lockdown certainly made them more prone to shame. Indeed, as Nussbaum points out, shame has its origins in bodily vulnerability (Nussbaum, 2004, p.116) and what we designate as shameful is defined against our common human vulnerabilities (e.g., mortality, illness, physical dependency). If anything, the conditions of occupation registered by Sartre indicate how such individual vulnerabilities become collective experiences in circumstances like lockdown. But vulnerability to shame can also push us towards shaming. Donald Nathanson makes clear with his ‘compass of shame’ (Nathanson, 1992) that the ‘attack other’ response is a common way that shame is bypassed or avoided, where one may lash out at others through an act of aggression or shame, as a displacement of one’s own vulnerability (Gilligan, 1999). In this way, the affective atmospheres of lockdown may have pushed individuals towards the shaming of others, a displacement of their own vulnerability through a form of aggressive agency.

## Conclusion

By April 2020, some ninety countries had instituted lockdowns, meaning more than 3.9 billion people were asked or ordered to stay at home. Such conditions should, seemingly, have produced uniform experiences of pandemic shaming, if Sartre’s point were universally applicable. As yet, no such uniformity has appeared. Certainly, a wide range of confounding factors may explain why no single form of pandemic shaming occurred: cultural, social, and infrastructural concerns meant that pandemic lockdowns could affect households on the same street differently, let alone people in different countries. But the case of pandemic shaming in the UK is particularly useful in illustrating the potential consequences of poor public health guidance when combined with the atmospheres of vulnerability generated in lockdowns and under occupations. Lockdowns (or occupations) are circumstances where individuals, because they are faced with their own bodily vulnerability, are more prone to feeling shame and are more likely to shame others. Policies that obscure the terms of these lockdowns, it seems, heighten this sense of vulnerability, and, concomitantly, shame proneness and the potential for shaming. Of course, clearer policies do not, in themselves, produce less shame or shaming. For Sartre, the problem of life under Occupation is not a lack of clarity about specific guidance; it is the proliferation of an atmosphere of distrust and shame whose connection to the actual German soldiers remains vague and indefinite. If the UK example shows the need for policies that are clear in their protocols, it is even more important that they be sensitive to their shame-producing potential.

In this article, we have established the circumstances that gave rise to a distinct form of pandemic shaming. We have shown how the role of the audience was key to the initial impulses to shame and to the shame backlashes that occurred when these shaming bids went wrong. Turning to Sartre’s account of the look gave us phenomenological tools for examining the agents at work in these circumstances. But we also returned this analysis to its historical context, to show corollaries between Sartre’s affective atmospheres under the Occupation and those generated during lockdowns. As a consequence, we can say that under lockdown conditions, particular self-other relations emerge that are predisposed to shame and shaming. For this reason, future planning about pandemic preparedness demands a shame-sensitivity attuned to phenomenological method.

## Footnotes

1 Hansard HC Deb 11 May 2020, vol 676, col 30. https://hansard.parliament.uk/commons/2020-05-11/debates/D92692B5-165B-4ACB-BC97-4C3F25D726EE/Covid-19Strategy.

2 It should be noted that the necessity of an audience in a shame experience is contested. Some thinkers argue that shame can be an entirely personal experience without the need for a ‘witness’ or ‘other’ to activate the shame emotion (Deonna et al., 2012).

3 This is something Sartre acknowledges in his later work. In his essay ‘Black Orpheus’, Sartre writes: “I want you to feel, as I, the sensation of being seen. For the white man has enjoyed for three thousand years the privilege of seeing without being seen. It was a seeing pure and uncomplicated; the light of his eyes drew all things from their primeval darkness. The whiteness of his skin was a further aspect of vision, a light condensed. The white man, white because he was a man, white like the day, white as truth is white, white like virtue, lighted like a torch all creation; he unfolded the essence, secret and white, of existence. Today, these black men have fixed their gaze upon us and our gaze is thrown back into our eyes. By this steady and corrosive gaze, we are picked to the bone” (Sartre, 1976, p. 7).

4 For instance, a woman may be actively shamed for not wearing a headscarf in a Muslim country, but may not feel the least bit of shame as a result if this is not a norm or value she shares. Nonetheless, examples like this are contested and there is a longstanding debate in the philosophy of emotions regarding whether shame is a heteronomous or autonomous emotion (Calhoun, 2004). While the first-person experience of shame is not a necessary outcome of shaming, we believe it is also the case that individuals can be made to feel shame through shaming, even when they do not agree with the norms or judgements by which they are being shamed.

5 A translator’s note from Sarah Richmond following the above quote reads: “It seems likely that Sartre is thinking of tactics used by the French Resistance in the Second World War” (note 31, 353). To give a sense of the chronology, the German Occupation of France officially ran from May 1940 to December 1944, and *Being and Nothingness* was published in 1943. Certainly the paranoiac inference of the look in rustling bushes, footsteps, opened shutters and twitching curtains recreates the claustrophobic conditions of Sartre’s dramatic works during continued German rule, like *Huis Clos* and *Les Mouches*. One might catalogue such references, quilting them together to historicize *Being and Nothingness* as a product of the Occupation’s atmosphere.
